# City-wide space-time patterns of environmental noise pollution in Kigali, Rwanda

**DOI:** 10.1088/1748-9326/ae1f2c

**Published:** 2025-11-24

**Authors:** Jean Remy Kubwimana, Sierra N Clark, James Nimo, Chantal Umutoni, Pacifique Karekezi, Barbara E Mottey, Claudette Nyinawumuntu, Samson Niyizurugero, Silas S Mirau, Pie-Celestin Hakizimana, Isambi S Mbalawata, Paterne Gahungu, Majid Ezzati, Allison F Hughes, Raphael E Arku

**Affiliations:** 1 School of Computational and Communication Science and Engineering, The Nelson Mandela African Institution of Science and Technology, Arusha, Tanzania; 2 African Institute for Mathematical Sciences Research and Innovation Centre, Kigali, Rwanda; 3 School of Health and Medical Sciences, City St George’s, University of London, London, United Kingdom; 4 Department of Physics, University of Ghana, Accra, Ghana; 5 Department of Environmental & Sustainable Engineering, State University of New York, Albany, NY, United States of America; 6 Department of Environmental Health Sciences, University of Massachusetts, Amherst, MA, United States of America; 7 Rwanda Environment Management Authority, Kigali, Rwanda; 8 Department of Mathematics, Faculty of Natural Sciences, Imperial College London, London, United Kingdom; 9 Department of Epidemiology and Biostatistics, School of Public Health, Imperial College London, London, United Kingdom; 10 Regional Institute for Population Studies, University of Ghana, Accra, Ghana; 11 MRC Centre for Environment and Health, School of Public Health, Imperial College London, London, United Kingdom; 12 Imperial Global Ghana, Accra, Ghana

**Keywords:** environmental noise, measurement, sensors, sub-Saharan Africa, East Africa, Rwanda, Kigali

## Abstract

As cities in sub-Saharan Africa become more crowded, noise pollution is also emerging as an important environmental concern, after air pollution. Yet, unlike air pollution, which is enjoying relatively more public attention, there is limited measurement data and policy efforts on environmental noise pollution. We followed a recent city-wide measurement approach used in Accra (Ghana) and characterized environmental noise patterns in Kigali, a contrasting city with very different topography and regulatory system than Accra to inform urban policy. We established 10 ‘fixed’ (yearlong) and 120 ‘rotating’ (weeklong) monitoring sites to capture both the temporal and spatial patterns in Kigali’s sound environment. The measurement occurred between November 2022 and December 2023, and samples were collected at 1 min interval, resulting in 5155 014 (3580 site-days) and 1190 620 (827 site-days) site-minutes of valid data from the fixed and rotating sites, respectively. The 130 monitoring sites covered a variety of geographic and land-use factors across diverse neighborhoods and sources. We computed several noise metrics, including 1 h (LAeq_1 h_), daily (LAeq_24 h_), day-time (*L*_day_), and night-time (*L*_night_). Daily noise (LAeq_24 h_) levels across the city ranged between 38 dBA and 85 dBA. Commercial, business, and industrial (CBI) and high-density residential (HD) communities experienced the highest noise levels, with some sites constantly above 70 dBA at day and 65 dBA at night. About 63% of our observed day-time values (up to ~72% in some areas) exceeded the Rwandan day-time standard (55 dBA) for residential areas, whereas 69% of the observed night-time values (up to 80% in some areas) exceeded the corresponding night-time standard (45 dBA). In Nyarugenge, the most urbanized district, as much as 75% of our site-days data exceeded day-time standard. However diurnal patterns throughout the city were similar, rising from ~5 am, peaking at about 8 am and plateauing until 6 pm before falling to their lowest at midnight. Overall, noise levels in the city did not vary much by day of the week, weekdays vs weekend, or dry vs wet seasons. Environmental noise in Kigali often exceeded both Rwandan standards and international guidelines, with residents in the city center district, CBI and HD areas at risk of higher exposure, and hence higher risk of adverse effects. Detailed assessment of the sources, at-risk population, and associated health effects may inform Rwandan’s environmental policy efforts and city initiatives in the face of the ongoing urban growth and densification.

## 1 Introduction

As socioeconomic opportunities continue to attract people to cities worldwide, urban dwellers are faced with the dangers posed by environmental hazards, including air and noise pollution [1–5]. Much like air pollution, urban environmental noise exposure has been shown to impact physical health and mental well-being [6–11]. Epidemiological studies, mostly investigating long-term population exposures to transportation noise in Europe, have shown robust associations with annoyance, sleep disturbance [12–15], and cardiometabolic health outcomes and risk factors [16–19]. However, there is limited epidemiologic studies in many places outside of Europe due to lack of high-resolution and city-scale exposure data [20–25]. As cities in developing countries become more crowded, noise pollution is also emerging as an important environmental concern, after air pollution [26–30].

Presently, sub-Saharan Africa (SSA) has the world’s fastest growing, youngest population, and the highest urban growth rate. Consequently, noise pollution is increasingly becoming a public health risk in growing cities [20, 31, 32]. While emerging measurement data have resulted in increasing resource mobilization and public campaigns to combat rising air pollution levels in SSA cities, similar efforts to manage urban soundscapes are limited. One key reason for this lack of public and policy engagement on noise pollution in the region is the scarcity of measurement data. The few measurement studies that exist have shown elevated noise levels that exceed international and national guidelines [31–35]. These studies have identified some urban sources that are similar to those in developed country cities (e.g. road traffic and aircraft) and others that are unique to SSA (e.g. loud music from informal businesses and religious activities in residential areas) [33, 36–39]. With such diversity of sources in fast-sprawling SSA cities amid weak regulatory framework, city-wide measurements are critical for understanding the magnitude and spatial patterns of noise pollution and identifying atrisk communities and populations to inform policy interventions [31, 39–43].

Considering the general lack of data in SSA, we developed and implemented in Accra (Ghana) a consistent and transferable protocol for generating rich environmental health data in urban SSA context, as part of the ‘Pathways to Equitable Health Cities’ project [43]. In Kigali, we implemented the Accra protocol to understand environmental noise patterns in a contrasting city with very different topography and regulatory system than Accra to inform policy as the city continues to expand and densify. Kigali, the capital of Rwanda and central hub for technology, is one of the few SSA cities with major government efforts to regulate noise and air pollution. These policy efforts include curfews, car-free days and zones, and a set time for closing non-essential services at night, all of which may help reduce noise pollution. Yet, no prior city-wide noise assessment exists for Kigali, which can provide broad baseline data on the patterns and neighborhoods at risk, and allow for the evaluation of the effectiveness of these policies. Here, we provide the first city-wide data on environmental noise in Kigali city.

## 2 Materials and methods

### 2.1 Ethical consideration

The study was approved by the Rwanda Environment Management Authority (REMA). The African Institute for Mathematical Sciences Research and Innovation Centre (AIMS-RIC) managed the field campaign. The original study protocols (implemented in Ghana) were approved by the Imperial College London and the University of Massachusetts Amherst.

### 2.2 Study area

**Figure 1:**
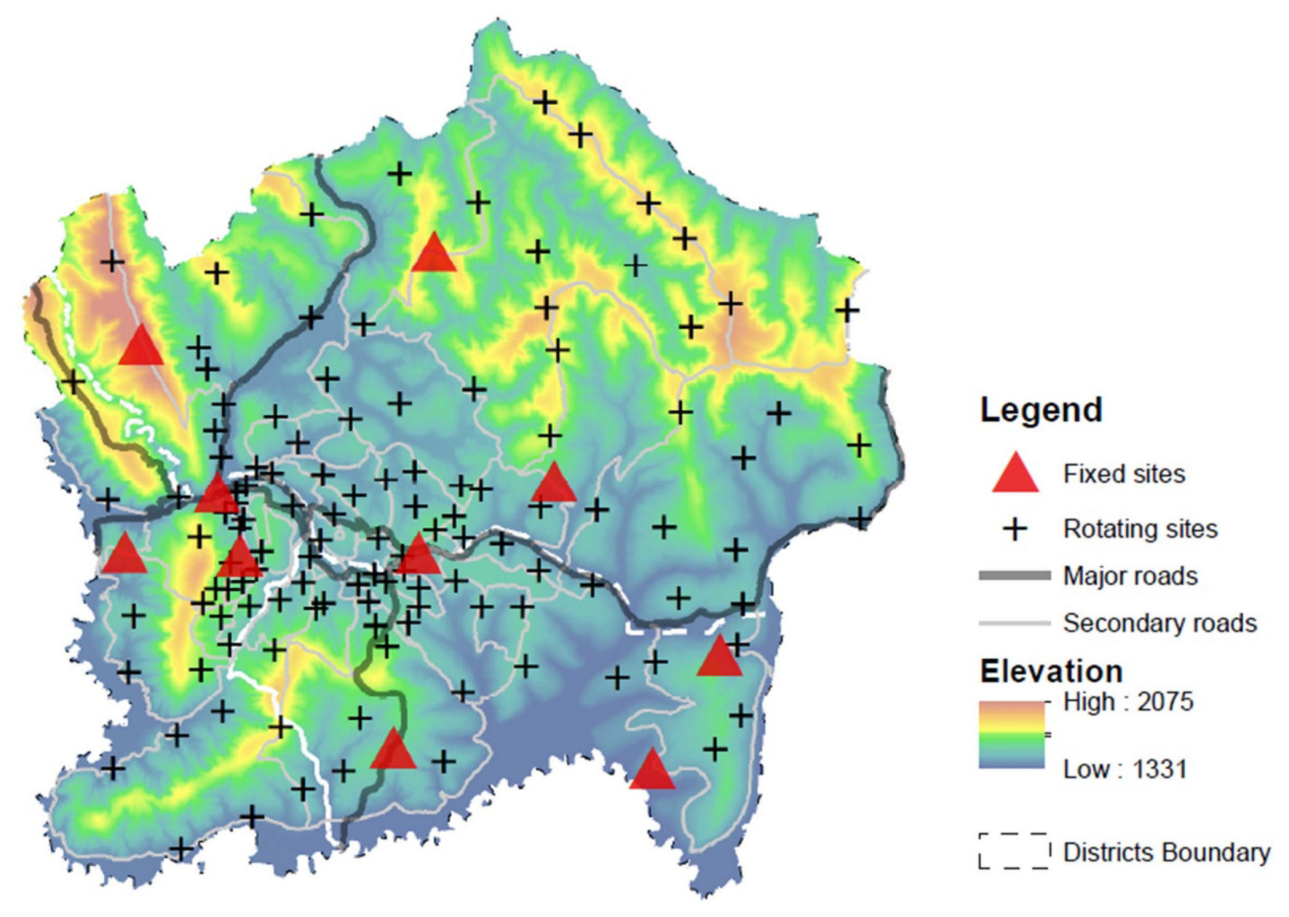
Map of Kigali city, showing elevation and monitoring sites. Fixed (yearlong) sites (*n* = 10) are represented by red triangles and rotating (weeklong) sites (*n* = 120) by black crosses. Elevation data (in meters) was sourced from open repositories and road networks were acquired from OpenStreetMap. See figure S1 for the boundaries of the three administrative districts: Gasabo, Kicukiro and Nyarugenge. Reproduced from OpenStreetMap and its contributors. CC BY-SA 2.0.

The Kigali city spans an area of 730 Km^2^ with ~1.75 million residents assembled in three administrative districts: Gasabo, Kicukiro and Nyarugenge [44]. Gasabo comprises primarily of agriculture land (background) and scattered mixed land use (areas with residential, commercial, and industrial activities), whereas Kicukiro is predominantly low- and medium-density residential communities. In contrast, Nyarugenge is the center and commercial hub of Kigali, and is characterized by dense commercial activities and heavy traffic. Kigali’s population is growing at an annual rate of 4.4% since 2012, nearly twice the national average of 2.3% [44], resulting in substantial socioeconomic, demographic, and land use changes [45, 46]. The built-up (more developed) areas increased from 48.6 Km^2^ in 2003–238.1 Km^2^ in 2023 [47]. The city’s topography is characterized by hills, valleys, and ridges, with peaks reaching up to >2000 m above sea level (figure 1). There are two rainy seasons in a year (March–May, and September–December), with dry season in June–August. As part of its green city and healthy initiatives, Kigali has instituted car-free days on the first and third Sundays of the month, where some roads are closed to vehicular traffic between 7 am and 10 am to allow open walking and cycling, and routine vehicle emission inspections [48]. There are also car-free zones with pedestrian and bicycle lanes to promote non-motorized transport.

### 2.3 Study design and site selection

**Figure 2:**
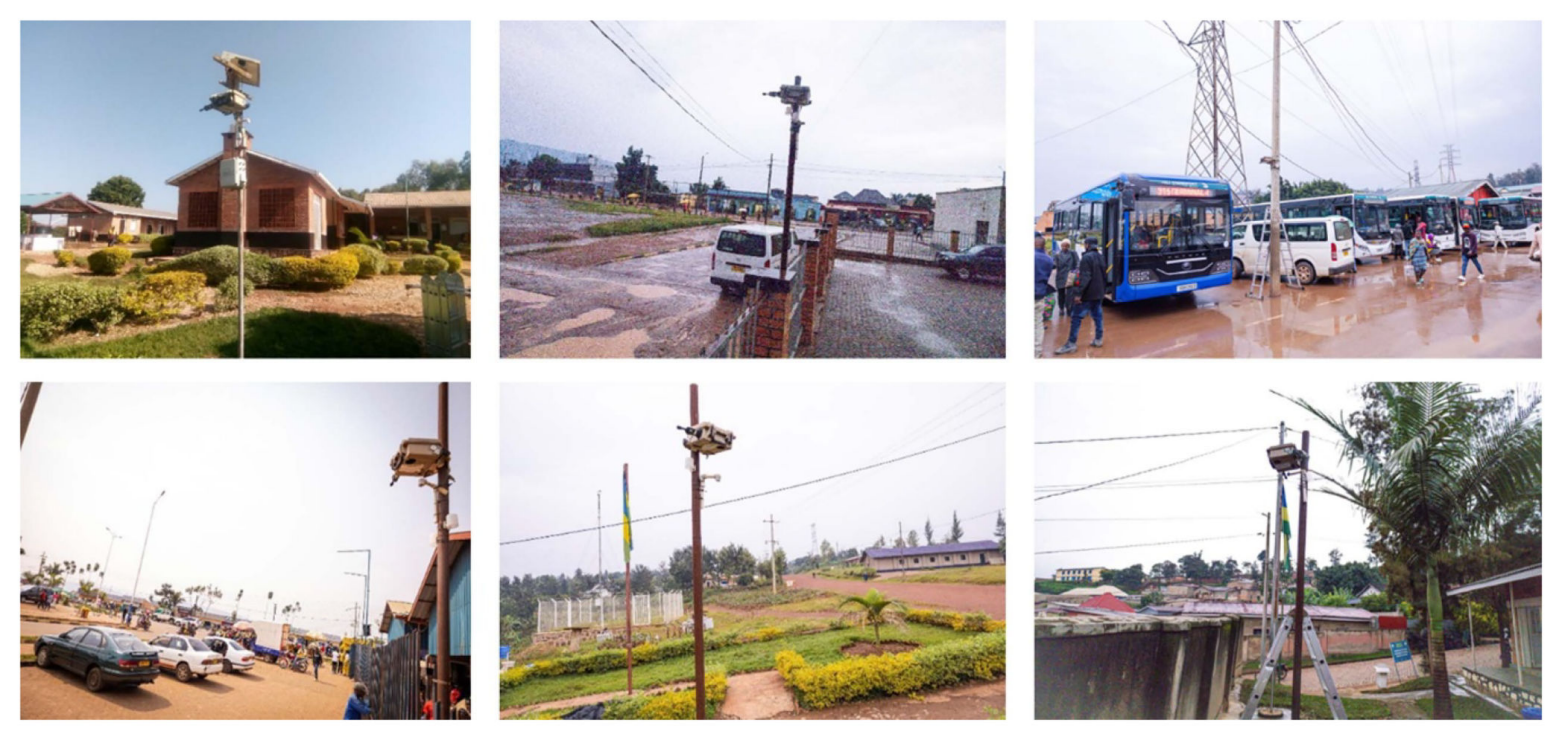
Examples of the measurement setup, equipment, and sites.

Our study design followed the measurement approach used previously in Accra, Ghana [43]. In Kigali, we established 10 ‘fixed’ (run continuously for a year; yearlong) and 120 ‘rotating’ (run continuously for a week; weeklong) monitoring sites to capture both the temporal and spatial patterns in the city’s sound environment (figure 1). The field measurements occurred between November 2022 and December 2023. The monitoring sites were selected based on land use and geographic factors, aiming to capture noise levels across diverse neighborhoods and areas of varied noise sources (figure 2). Detailed site-specific information was documented using standardized log forms. Each site was then classified as one of four land use categories as defined by Kigali’s City government’s 2020 Master Plan: commercial, business, and industrial (CBI, *n* = 19 [4 fixed and 15 rotating]); high-density residential (HD, *n* = 28 [1 fixed and 27 rotating]); medium/low-density residential (LD, *n* = 49 [2 fixed and 47 rotating]); or background/peri-urban (BG, *n* = 34 [3 fixed and 31 rotating]) [49]. CBI areas cover places with commercial, business and industrial activities and along major roads with higher potential for noise. HD represents neighborhoods with dense and lower-income populations, narrow roads with substantial traffic with chances of noise from mix of traffic and human activities, whereas LD are communities with sparse to moderate concentration of dwellings and populations with higher incomes, wider roads and lower traffic. Compared to other land use types, BG areas include places with high green or open space and minimal traffic influence.

### 2.4 Sound level measurement

We captured maximum (L-Max dBA), equivalent continuous (LEQ dBA), and minimum (L-Min dBA) environmental sound levels using a Noise Sentry sound level meter (SLM) (https://convergenceinstruments.com/) with a dust-protected type I MEMS microphone [43, 50]. The sensors were mounted on a wind and rain protective box on metal poles at ~4 m above ground and ~2 m away from any direct and surrounding noise sources and facades (figure 2). The data were recorded at one-minute intervals [51]. The Noise Sentry SLM sensors were validated against a Type I industry-standard instrument (DUO 01 dB), and showed very high agreement with the mean and median second-by-second differences of −0.42 and −0.38 dBA, respectively [41, 43]. During our monitoring campaign, the sensors were first collocated and tested in the lab and found high between- and within-sensor consistency. In the field, we collected duplicate samples at 33% (1 in 3) of the rotating sites (figure S6). The absolute median and mean minute-by-minute difference between the duplicate and main samples were 0.89 (−0.02) and 1.3 (0.03) dBA, respectively.

### 2.5 Data management and noise metrics

We inspected the data for implausible readings and removed 0.32% minute-by-minute data points that were unrealistically low (<20 dBA); none were unrealistically high (>120 dBA) [52, 53]. Our final analysis included 5155 014 (3580 site-days) and 1190 620 (827 site-days) site-minutes of data from the fixed and rotating sites, respectively. For each site, we computed 1 h (LAeq_1 r_), daily (LAeq_24 h_), day-time (*L*_day_), and night-time (*L*_night_) equivalent continuous noise levels, along with day-time and night-time intermittency ratios (IR_day_, IR_night_) (%) [41, 54]. The IR is defined as the ratio of event-based sound energy to the total sound energy during a given measurement period, and expressed as percentage [16, 55, 56]; where 0% indicates no distinct noise events above the background, and 100% indicates all noise energy comes from individual events [16, 31]. In this study and similar to past studies [41, 54], we used a threshold of +3 dBA above the *L*_eq,T,tot_ (offset *C* = 3 dBA). Following the Rwandan acoustic standards, we defined day-time as 06:00–20:59 and night-time as 21:00–05:59 [57].

Both sound level and event metrics were summarized using the median and interquartile range (IQR) as a measure of central tendency and spread/variation as the measured data were not normally distributed. Using data from the fixed sites, we examined temporal patterns in the noise metrics across various time spans, including diurnal, days of the week, weekdays vs weekends, and rainy vs dry season. Spatial patterns were evaluated across the four land use categories (CBI, HD, LD, and BG) using data from both the fixed and rotating sites. We also compared noise levels across the three administrative districts. Further, we examined the percent of sites/data that surpassed the Rwandan standard for residential areas [57] and the World Health Organization (WHO) 2018 European Environmental Noise guidelines [58, 59]. Data analysis and visualization were performed using R (R version 4.5.0).

## 3 Results

### 3.1 Spatial patterns in noise level and event metrics

The median (IQR) daily (LAeq_24 h_) across all 120 rotating (weeklong) sites was 54.7 (IQR: 50.5, 58.7) dBA, with CBI areas ~5 dBA louder (i.e. ~three times more intense) than BG areas (table 1). Day-time (*L*_day_) median noise levels ranged from 54.7 (IQR: 51.4, 59.1) dBA and 54.6 (IQR: 51.4, 58.2) dBA at BG and LD areas, to 57.2 (IQR: 54.3, 61.9) dBA at HD, and 59.3 (IQR: 56.0, 65.5) dBA at CBI areas. There was about 8 dBA drop in the overall noise levels between day-time and night-time (56 vs 48 dBA), which is ~6-fold reduction in sound energy and nearly half the perceived loudness. The highest night-time (*L*_night_) median noise levels of 53.3 (IQR: 50.8, 59.8) dBA occurred at CBI areas, and they were about the same as the lowest day-time levels at LD and BG areas. Within land-use categories, BG sites experienced the highest day- and night-time variability (~10 dBA), compared to ~6.0 dBA across the other land use categories. BG and LD residential areas also had the highest median IRs in both the day and at night (>50%), whereas CBI areas were below 50%. In general, land use areas with higher sound levels had lower IRs (table 1). The yearlong data from the individual fixed sites also followed the same land use pattern, with the highest median daily (LAeq_24 h_) level of ~70 dBA observed at a traffic dominant site whereas the lowest levels were recorded at peri-urban sites (table S1). Consequently, IRs at traffic and CBI sites were substantially lower than IRs at other land use sites (table S1).

**Figure 3:**
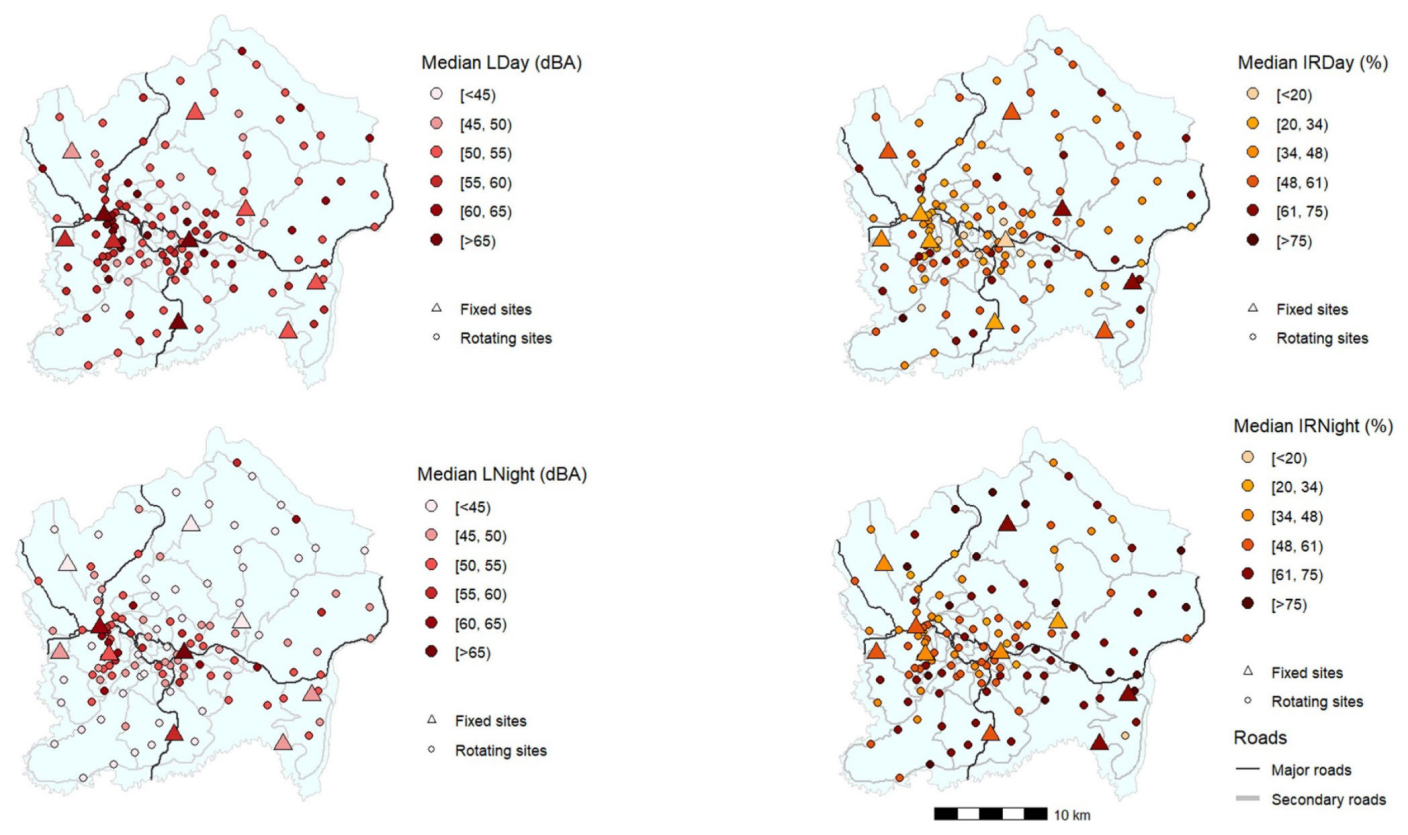
Spatial distribution of day-time (L_day_) and night-time (L_night_) median noise levels, along with the intermittency ratio during day-time (IR_day_) and night-time (IR_night_) for both rotating and fixed monitoring stations. Triangle markers represent yearlong fixed sites and circle markers indicate rotating sites.

Figure 3 shows the spatial distribution of the measured noise (*L*_day_, *L*_night_, IR_day_, IR_day_) levels across the entire city. In general, measurement sites in the more remote areas with scattered settlements had the lowest noise levels in comparison to densely populated and central areas closer to residences, major roads, and business activities. The median day-time (*L*_day_) at measurement sites along major roads ranged 54.5–63.2 dBA compared to 52.4–57.8 dBA in residential areas. A similar pattern was observed at night-time between traffic and residential areas, except that the levels were about ~6 dBA lower at night.

**Figure 4:**
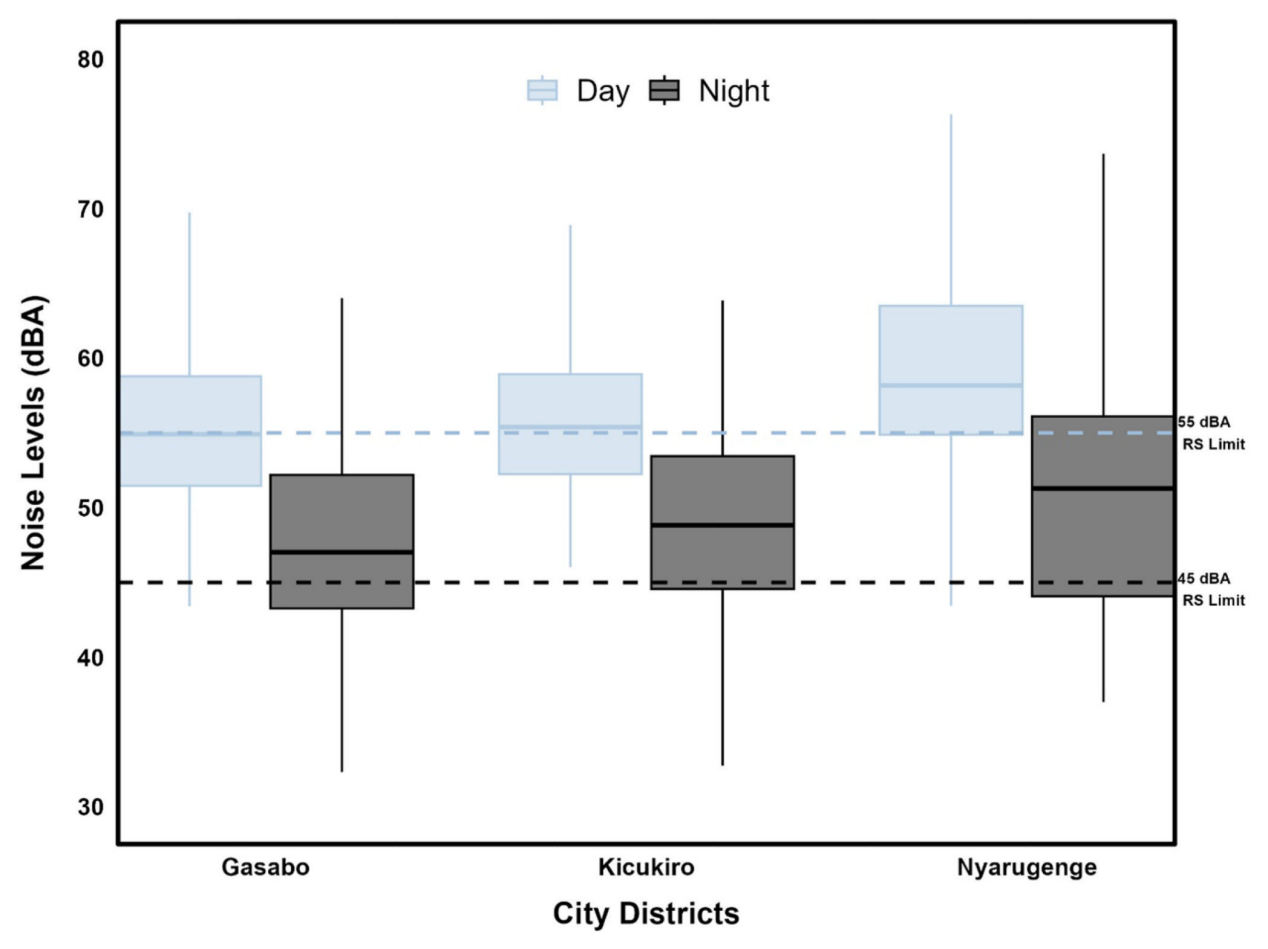
Box plots displaying the distribution of day-time (*L*_day_) and night-time (*L*_night_) noise levels (for rotating data) by districts of Gasabo (*n* = 57), Kicukiro (*n* = 32), and Nyarugenge (*n* = 31). The dashed lines represent the Rwandan national noise standards of 55 dBA during the day and 45 dBA at night for residential areas.

Across the three administrative districts (figure S1), both day-time (*L*_day_) and night-time (*L*_night_) noise levels were often above the national standards for residential areas (55 dBA for day and 45 dBA for night), particularly in the busiest Nyarugenge district where 75% of the day-time values exceeded the day-time limit, compared to ~50% in the other two districts (figure 4 and table 2). While over 60% of the data in all three districts exceeded the night-time limit, Gasabo district was relatively quieter than both Kicukiro and Nyarugenge, which was the loudest even at night. Regardless of the district, there were also strong within-district differences, which were driven by land use activities.

### 3.2 Temporal patterns in noise level and event metrics

**Figure 5:**
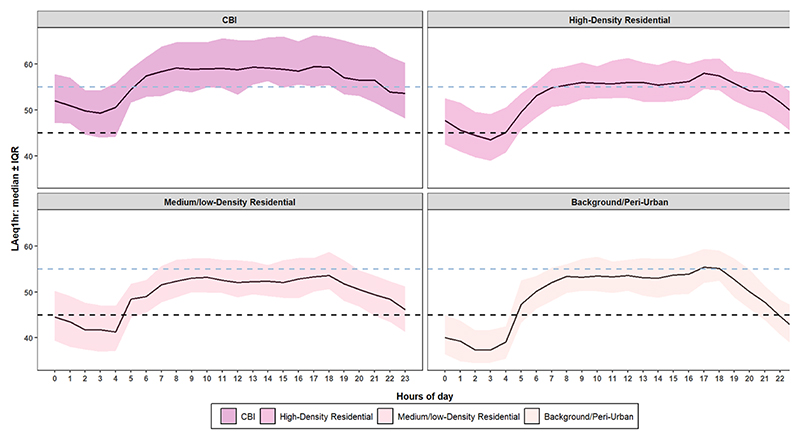
Diurnal variation of hourly noise (LAeq_1 h_) levels across time of day and site types. Trend lines show hourly median noise levels, with shaded areas representing the 25%–75% percentiles. The horizontal dashed lines are the 45 and 55 dBA Rwanda day- and night-time limit for residential areas respectively.

All sites and site-types exhibited similar diurnal patterns in noise level across time of day (figure 5). Median hourly noise (LAeq_1 h_) levels rose from around 5 am, peaked at about 8 am and plateau until 6 pm when they began to fall to their lowest between midnight to 4 am (figures 5 and S2). Between 6 am and 8 pm, the median hourly (LAeq_1 h_) levels at CBI and HD areas exceeded the Rwandan day-time limit of 55 dBA for residential areas. This pattern existed also at the fixed sites when the data was aggregated across the full year, particularly sites dominated by traffic and commercial activities. Overall, ~44% of the hourly (LAeq_1 h_) data at day-time hours exceeded the 55 dBA threshold for residential areas, whereas 54.3% of night-time data exceeded the 45 dBA limit.

For day versus night, the median day-time noise levels (*L*_day_) at CBI sites was 58.8 dBA compared to 52.4 dBA at night. They were 55.8 vs 48.0 dBA at HD areas, 52.4 vs 45.4 dBA at LD neighborhoods, and 53.3 vs 41.5 dBA at BG sites. During the day, ~72% of the data at CBI and ~57% at HD exceeded the national limit, compared to only ~36% in BG sites. At night, 82% of the CBI data exceeded the national night-time limit of 45 dBA, followed by 66% at HD, 52% at LD, and 34% at BG. In general, CBI consistently showed the highest exceedance rates for both day and night limits whereas BG had the lowest exceedance rates. Although car-free Sundays only occur on certain roads (same road) on the first and third Sunday of the month and for a limited time (07–10:00) only, we observed ~3 dBA reductions in the hourly (LAeq_1 h_) levels in HD areas, representing a 50% reduction in the sound energy. We found no clear impact of the car-free policy within other land use groups.

**Figure 6:**
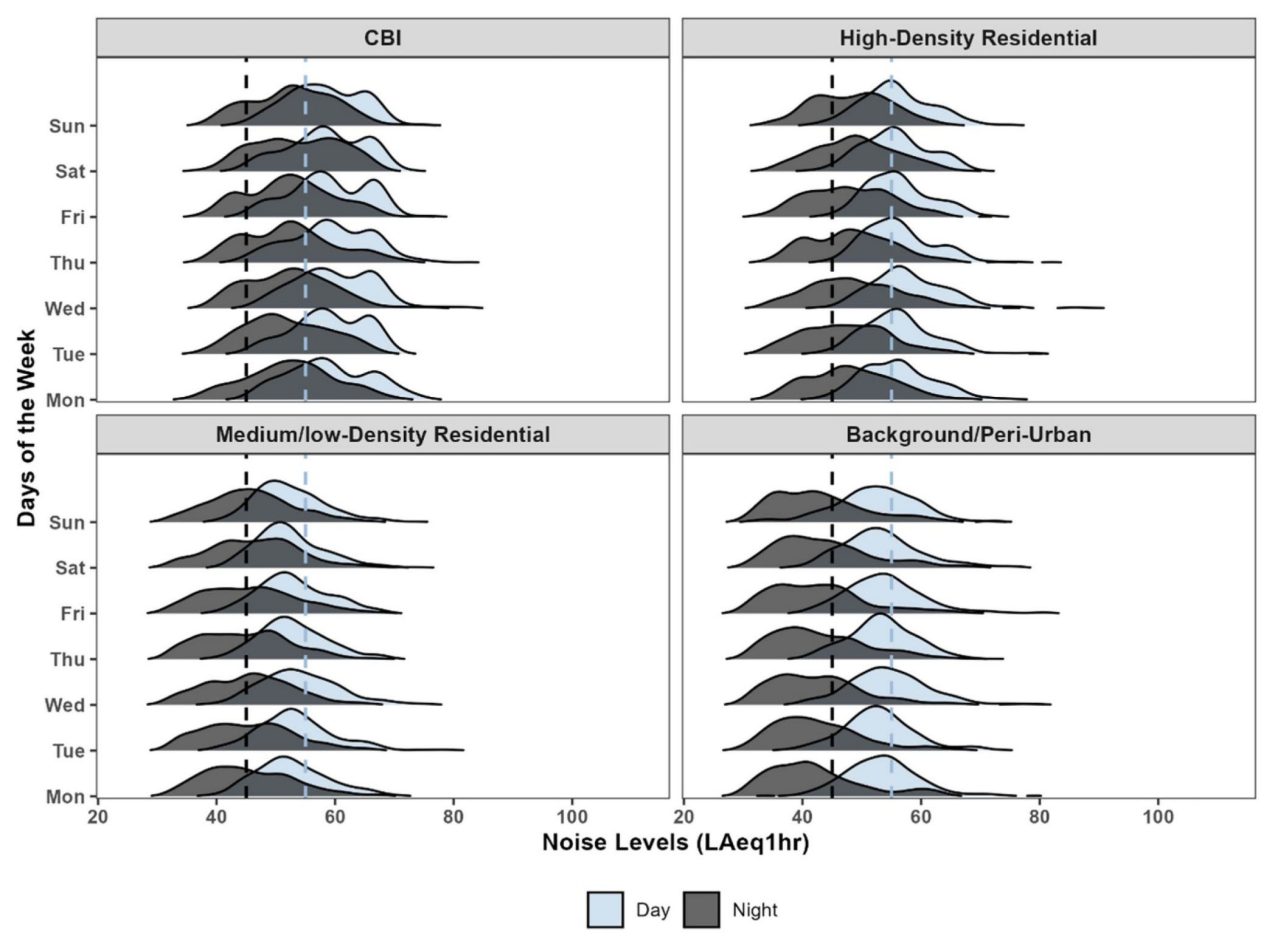
Distribution of day- (*L*_day_) and night (*L*_night_) -time noise levels by site types. Day-time is defined as 06:00–20:59 and night-time as 21:00–05:59 according to Rwanda Standard. The vertical dashed lines represent night-time (45 dBA) and day-time (55 dBA) national permissible limit for residential areas, respectively.

**Figure 7:**
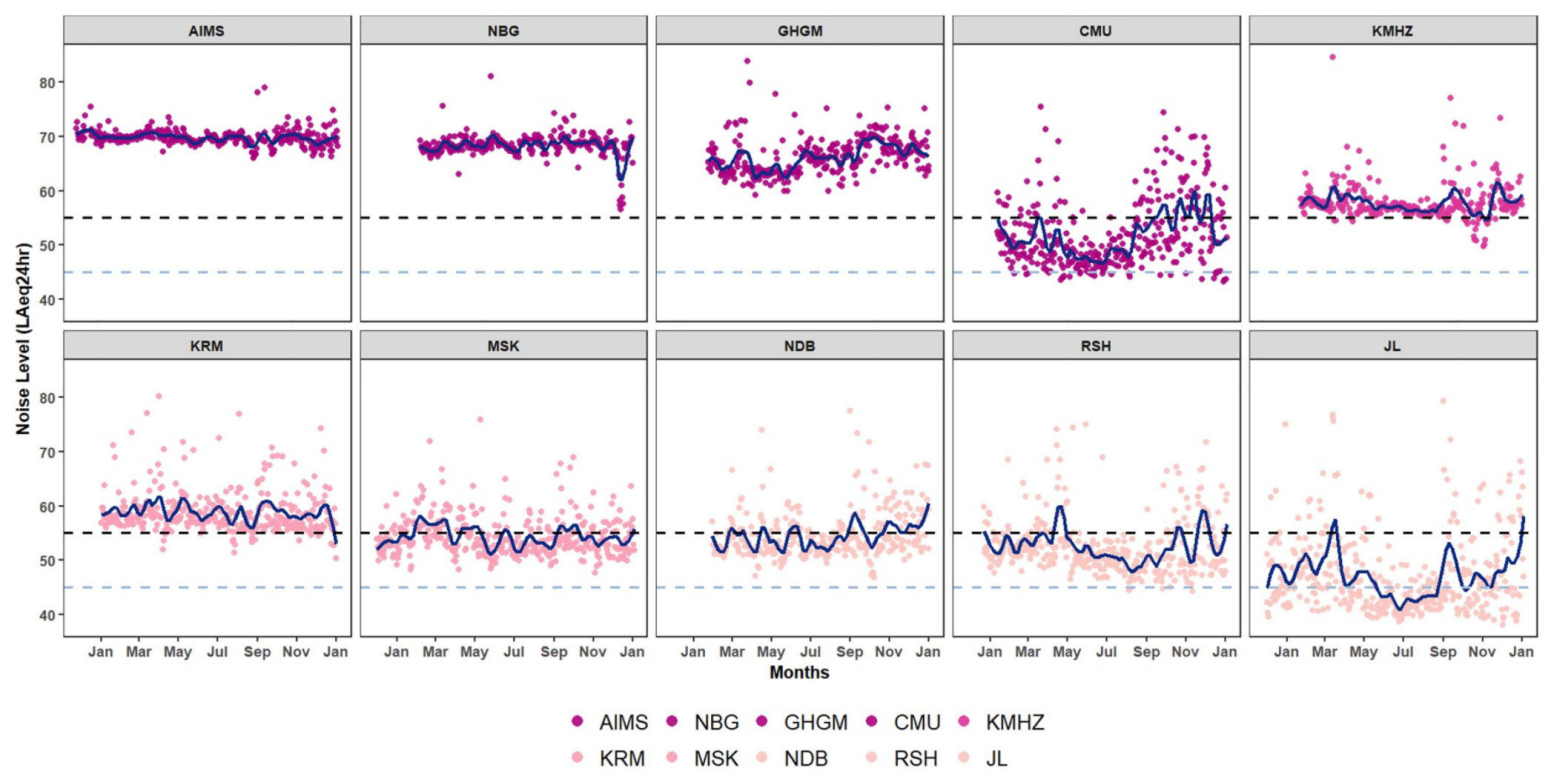
Timeseries of daily (LAeq_24 h_) noise levels across the full measurement year at the 10 fixed sites. The figure presents site-specific differences, temporal trends and seasonal variations. Commercial, business, industrial (AIMS, NBG, GHGM, CMU); high-density residential (KMHZ); medium/low-density residential (KRM, MSK) and background/peri-urban (NDB, RSH, JL). The two dashed lines represent the day-time (55 dBA) and night-time (45 dBA) national permissible limits for residential areas.

Over the one-year measurement period, daily (LAeq_24 h_) noise levels ranged from 38.1 dBA at a background site to 84.6 dBA at a HD residential site. By days of the week, the median noise levels during weekdays and weekends were nearly identical for each land use type (figures 6 and S3). It was 56.7 vs 57.0 dBA at CBI sites and 49.6 vs 50.5 dBA at medium/low-density residential and background/peri-urban sites. By season, the range of median daily (LAeq_24 h_) values (low vs high) were similar for dry (44 vs 70 dBA) and rainy (46 vs 70) dBA seasons. However, LD and BG fixed sites (e.g. RSH and JL) demonstrated noticeable month-to-month fluctuations. Throughout the year across the individual ten fixed sites, daily (LAeq_24 h_) noise levels at sites dominated by traffic (AIMS and NBG) consistently averaged around 70 dBA (figures 7 and S7). Five of the 10 sites located in CBI and HD areas had daily (LAeq_24 h_) levels above the day-time limit, while LD and BG sites mostly recorded levels above night-time limits and sometimes above day-time limits (figure 7).

## 4 Discussion

Unlike most fast-growing cities in SSA, Kigali has strong policy and regulatory framework around transport and commercial and residential activities with the goal of curbing environmental exposures [57, 60]. The city enforces routine vehicle emission inspections and residential night-time curfews on certain activities to reduce noise and air pollution accordingly. Its green city and healthy initiatives include car-free zones and days to promote non-motorized transport and reduce pollution. Yet, it is unknown what the levels are throughout the city and how they vary across various communities and over time. Such information is relevant for assessing the effectiveness of these policy interventions over time and informing future city planning goals. Following a large-scale citywide year-long measurement campaign, we found that environmental noise levels in Kigali were strongly patterned by land-use features, with the highest levels at traffic-dominant CBI and HD residential communities. Across the city, day-time and night-time noise levels often exceeded the Rwandan national standards and international guidelines for residential areas, particularly in Nyarugenge district, CBI, and HD neighborhoods. We also found a large difference between day-time and night-time levels, regardless of the landuse type. However, the highest night-time levels at CBI areas were about the same as the lowest day-time levels recorded at LD and BG areas, which also experienced the largest day- and night-time variability. Further, we found that areas with the highest sound levels had the lowest IRs, suggesting consistent continuous high background noises at these sites. Overall, we observed no significant differences across days of the week, between weekdays vs weekend, or dry vs wet seasons.

Our study was modeled after a previous one conducted in Accra, Ghana, with the aim of generating rich environmental health data in an urban SSA context [43]. But unlike Accra, Kigali is smaller in area, population, and traffic density; has a topography consisting of steep hills and valleys with a greener landscape; and with stronger environmental regulation and sustainability goals [61, 62]. Consequently, environmental noise levels recorded in Accra were slightly higher than in Kigali [31, 41]. Despite the above key differences between the two cities, our findings of the spatial and temporal patterns are consistent with the Accra data. Like Accra, we found higher noise levels, as expected, in areas with CBI activities and in densely populated residential neighborhoods, exceeding local standard and international guidelines [36, 37, 41, 63–65]. Residents living in the more urbanized and densely populated districts of Kicukiro and Nyarugenge, constituting ~50% of Kigali’s population, are at risk of higher exposure, and hence higher risk of associated adverse effects [66].

Just as Accra, we found no seasonal and week-days variations in the Kigali’s soundscape, except for diurnal (day vs night) differences that were in themselves also impacted by land use factors. Kigali also experienced much larger day-/night-time differences, which could be attributed to its night-time residential noise control policies and/or lesser night-time economic/social activities than seen in Accra. In Kigali, we found that BG and LD residential areas in general had higher IRs (event-based sound energy, >50%) both in the day and at night compared with HD and CBI areas. This is likely because the average baseline noise levels in these areas are lower, and therefore, intermittent and distinct noise events are more detectable. The Accra study [41] found slightly lower IRs than what we recorded in Kigali.

Studies of relatively smaller scope and duration in other SSA cities (e.g. Johannesburg, South Africa; Ibadan, Nigeria; Nairobi, Kenya; and Iringa, Tanzania) also found the same spatial patterns of environmental pollution across land use factors [20, 63, 67–71], and the levels far exceeded international guidelines. Studies from major cities in North America (e.g. Atlanta, Los Angeles, and New York) [72], Europe (e.g. Paris, London and Amsterdam) [73] and Asia (e.g. Doha, Guangzhou and Seoul) [74–76] have also documented variations in environmental noise levels with respect to land use patterns. In American and European cities, elevated noise levels are driven primarily by transportation (road, rail, and aircraft) and industrial activities. In Kigali, noise levels are equally high in traffic congested areas and commercial districts in addition to densely populated residential areas [77–79]. Generally, the levels recorded in Western cities due to transportation are higher than observed in Kigali, which is likely due to Kigali’s lower traffic volume and strong speed control policy (speed limits are tightly controlled/enforced with speed cameras in Kigali). However, the share of Kigali’s population that live in areas exceeding regulatory standards and health guidelines is likely higher than in Western cities.

The WHO has developed a set of guidelines over the years to limit public health impacts of exposure to noise pollution from environmental sources, with the most recent update in 2018 [59]. A number of SSA countries, including Rwanda, Ghana, Tanzania, and South Africa, have also established national limits to safeguard public health, but large-scale monitoring efforts to support compliance and health studies are generally lacking [20, 21, 41, 80]. Where they exist, as has been shown in Accra (and now Kigali), several local communities are not meeting these requirements. Between 36%–72% (day-time) and 34%–82% (night-time) of our observed noise data across different land use categories in Kigali city exceeded Rwanda’s standard for residential areas (55 dBA for day-time) and (45 dBA for night-time). Those living in densely populated neighborhoods and commercial districts are at higher risks of exposure [81] and potential adverse impacts like annoyance and disturbances in sleep quality and quantity [78, 82, 83].

We observed systematic diurnal patterns in noise levels across the city, rising early morning and peaking at and plateauing between 8 am and 6 pm. This is consistent with patterns of rush hours in a typical 9 am–5 pm working hours and peaks with traffic and commercial activities. Public transportation during this period is characterized by traffic congestion from combination of diesel buses, motorcycle taxis, and taxicabs. Thus, the substantially consistent higher day-time noise in CBI and HD residential areas may be due to traffic movements, loudspeakers used for advertisement, and sounds from local bars, exposing residents to levels of health concern. However, Rwanda’s policy of restricted loud activities and noise emissions during night-time (e.g. closure of clubs/-bars by 1 am on weekdays and 2 am on weekends) may be contributing to the substantially lower night-time noise across board, although many communities still experience night-time noise levels above the local standard. To promote the wellbeing of residents, the city of Kigali implemented noise pollution related mechanisms such as quiet and free zones, time-based noise restrictions, and urban planning regulations [84]. There is an indication that Kigali’s car-free hours on the first and third Sundays of the month may be reducing noise levels during that time, especially in densely populated residential areas, and needs to be expanded to achieve city-wide impact. Though the 3 dBA seems small and may even be barely noticeable to most people, it represents a 50% reduction in the sound energy reaching their ears, which can meaningfully reduce risks of hearing loss, annoyance, or stress-related health effects over longer periods of exposure. Thus, expanding these policies across board will benefit Kigali residents. Further, the observed higher IR in relatively quieter LD and BG areas could indicate more irregular noise patterns, which likely adds to the effect of noise exposure on health [55].

Though consistent with Accra, our observed lack of seasonal and weekdays differences contrasts with most studies in North American and European cities that found higher noise on weekdays [85–88]. This could be explained by regional variations in traffic patterns, urban infrastructure, and daily patterns of social and economic activities. Rapidly growing SSA cities like Kigali and Accra may be experiencing a more balanced commercial and commuter traffic throughout the week than in North America and Europe, where weekday peaks reflect more structured work schedules and commuter traffic.

As the city forges towards its ambition of growing and densifying within its unique physical layout of varying topography and greenery, it must combine timely data, city planning, and regulation to meet the growth/densification ambition, and associated mobility needs, and yet minimize environmental impacts like noise. To ensure urban change enhances health, well-being and sustainability, solutions may well include an effective public transport system that connects the city but is less polluting. One such example could be aerial tram/cable-car systems, which can work well for Kigali’s topography and reduce both traffic congestion and pollution. Cities like La Paz (Bolivia) [89, 90], Mexico City (Mexico) [91],Medellin City (Colombia) [92], Portland (USA), and Toulouse (France) [93] have incorporated aerial tramways or cable cars into their public transportation system and have been shown to reduce environmental pollution. Further, including the use of hybrid and fully electric buses in the public transportation system can also reduce noise pollution, particularly when combined with speed control and paving of road surfaces with low-noise materials. This is timely as Rwanda looks to restrict the importation of old vehicles.

### 4.1 Strengths and limitations

We employed a unique study design that allowed us to capture in detail the space-time variations in sound levels across an entire city. A major strength of our study is its large-scale, citywide and year-long measurement campaign involving >6.3 million site-minutes (4407 site-days) of data, enabling an in-depth analysis of spatiotemporal variations in environmental noise across diverse land-use settings. By capturing data from a broader range of residential, commercial, and mixed-use locations, we provide valuable insights into how specific urban features and human activities influence noise pollution. These findings offer crucial evidence to guide effective policy interventions and urban planning aimed at mitigating noise and improving public health.

As a limitation, we did not capture audio to objectively assess contributions from specific sound sources (e.g. traffic, loudspeakers, and nightlife establishments). Consequently, we cannot quantify the relative contributions of different noise generators or confirm when and where certain sources dominate, though we have inferred potential source influence through land use patterns. Additionally, while the study spanned a year and covered multiple seasons, more in-depth assessments (e.g. holiday effects, or variations in socio-economic activities) at targeted locations could enrich our understanding of noise dynamics. Despite these constraints, this work lays a strong foundation for the ongoing efforts to characterize and address environmental noise pollution in Kigali and other fast-growing SSA cities.

SSA cities that are looking to implement similar study design must be aware of a few key logistical challenges during the data collection, including sensor placement and access to optimal locations that reflect the city’s land use patterns. Also, quantifying the relative contributions from specific sources in SSA’s complex urban environment using combination of sound meters and audio will be critical for targeted policy interventions. Further, involvement of relevant local and national government agencies in the study design and interpretation of the results will be important for policy uptake and use of the data.

## 5 Conclusion

Like many SSA growing cities, Kigali faces environmental challenges, including noise pollution, which poses health risks to its residents. Yet, like other cities, Kigali lacked consistent city-wide noise to support policy efforts. We demonstrated in Kigali the successful transfer of our Accra protocol, which was designed generate rich environmental health data in urban SSA context. Our data show that there are a lot of areas, including residential communities in Kigali that far exceed the noise standards set by Rwanda and the WHO guideline. The findings call for targeted policies to achieve quieter, and hence healthier, living environment for Kigali residents in the face of the ongoing urban expansion.

## Supplementary Material

Supplementary material for this article is available online

Supplementary Information

## Figures and Tables

**Table 1:** Median (IQR) noise level and event metrics across land use categories.

Sites and site-types (*n*)	Noise metric (dBA)			Event metric (%)	
	LAeq_24h_	*L*_day_	*L*_night_	IR_day_	IR_night_
All rotating sites (120; 826 site-days)	**54.7 (50.5, 58.7)**	**56.1 (52.1,59.8)**	**48.2 (43.8, 53.6)**	**46.0 (30.5, 60.8)**	**55.4 (37.8,70.9)**
*Background/peri-urban (BG): (31)*	52.9 (49.6, 57.6)	54.7 (51.4, 59.1)	44.5 (41.7, 48.8)	51.1 (40.0, 62.2)	61.5 (43.5, 74.6)
*Medium/low-density residential (LD): (47)*	53.4 (50.1, 57.2)	54.6 (51.4, 58.2)	48.0 (43.4, 52.4)	52.1 (35.5, 63.6)	59.0 (40.3, 75.1)
*High-density residential (HD): (27)*	55.7 (52.6, 60.3)	57.2 (54.3, 61.9)	50.2 (45.3, 54.8)	37.8 (25.0, 53.8)	49.4 (33.2,61.3)
*Commercial/Business/Industrial (CBI): (15)*	57.8 (54.4, 64.3)	59.3 (56.0, 65.5)	53.3 (50.8, 59.8)	34.2 (22.8, 44.8)	49.3 (34.1, 57.7)
All fixed sites (10; 3584 site-days)	**56.8 (51.7, 66.6)**	**58.1 (53.1,68.2)**	**49.2 (44.0, 57.3)**	**46.8 (28.8, 65.2)**	**51.6 (39.7,66.6)**
*Background/peri-urban (BG): (3)*	51.2 (47.1, 54.2)	52.7 (48.5, 55.7)	42.9 (38.4, 45.5)	59.5 (50.6, 70.8)	63.0 (47.2, 75.6)
*Medium/low-density residential (LD): (2)*	56.3 (53.3, 58.5)	57.9 (54.8, 60.1)	48.3 (47.1, 50.1)	52.6 (40.9, 70.8)	63.4 (53.1, 76.6)
*High-density residential (HD): (1)*	57.1 (56.2, 58.3)	58.1 (57.2, 59.3)	53.8 (53.0, 54.7)	31.9 (24.6, 42.3)	46.6 (41.3, 52.4)
*Commercial/Business/Industrial (CBI): (4)*	67.7 (61.9, 69.4)	69.4 (63.6, 70.8)	60.0 (54.0, 65.5)	28.2 (15.5, 49.1)	42.6 (28.5, 51.8)

LAeq_24 h_: A-weighted equivalent continuous 24 h noise level; *L*_day_ and *L*_night_: a-weighted equivalent continuous noise level in the day- and night-time; IR_day_ and IR_night_: day- and night-time IRs (%). Commercial/Business/Industrial (CBI) are places with commercial, business and industrial activities and along major roads; HD residential represent neighborhoods with dense populations, narrow roads, and high biomass use; medium-/low density residential (LD) are neighborhoods with sparse to moderate concentration of dwellings and populations with wide roads and low biomass use; and background/peri-urban (BG) areas are places with high green or open space and minimal traffic influence. See table S3 for detailed number of samples by site-days and site-hours for each noise metric/event and land use type classifications.

**Table 2:** Population characteristics and noise levels across districts.

District	Gasabo	Kicukiro	Nyarugenge
Percent of Kigali population (%)	50.4	28.2	21.4
Population density (people/km^2^)	20,56	2,944	2,830
Noise pollution (IQR)			
*Day-time (L_day_)*	54.9 (51.5, 59.0)	55.4 (52.2, 58.9)	58.2 (54.9, 63.5)
*Night-time (L_night_)*	47.0 (43.3, 52.2)	48.8 (44.6, 53.4)	51.3 (44.1, 56.1)
*Daily (LAeq_24h_)*	53.4 (50.0, 57.1)	54.3 (50.8, 57.8)	56.9 (53.4,61.8)
Event metrics			
*IR_day_*	44.8 (32.3, 59.6)	50.0 (31.9, 61.8)	44.7 (28.7,61.7)
*IR_night_*	55.4 (35.3, 71.4)	58.3 (38.9, 74.6)	53.0 (38.9, 64.5)
*IR_daily_*	1.91 (0.40, 5.64)	2.74 (0.77, 8.60)	1.39 (0.19, 4.53)

## Data Availability

The data that support the findings of this study are available upon reasonable request from the authors.
