# Supplementary material for: City-wide space-time patterns of environmental noise pollution in Kigali, Rwanda
Source: Environ Res Lett. Author manuscript; Available in PMC 2025 Dec 11. (PMC7618465; doi:10.1088/1748-9326/ae1f2c)
Supplement: Supplementary Information [file EMS211169-supplement-Supplementary_Information.docx]

**Supplementary Information**

**City-wide space-time patterns of environmental noise pollution in Kigali, Rwanda**

Jean Remy Kubwimana^1,2^, Sierra N. Clark^3^, James Nimo^4,5^, Chantal Umutoni^1,2^, Pacifique Karekezi^1,2^, Barbara E. Mottey^6^, Claudette Nyinawumuntu^2^, Samson Niyizurugero^2^, Silas S. Mirau^1^, Pie-Celestin Hakizimana^7^, Isambi S. Mbalawata^2^, Paterne Gahungu^2,8^, Majid Ezzati^9-13^, Allison F. Hughes^4^, Raphael E. Arku^6,^ *

^1^School of Computational and Communication Science and Engineering, The Nelson Mandela African Institution of Science and Technology, Arusha, Tanzania

^2^African Institute for Mathematical Sciences Research and Innovation Centre, Kigali, Rwanda

^3^School of Health and Medical Sciences, City St George’s, University of London, London, UK

^4^Department of Physics, University of Ghana, Accra, Ghana

^5^Department of Environmental & Sustainable Engineering, State University of New York, Albany, USA

^6^Department of Environmental Health Sciences, University of Massachusetts, Amherst, MA, USA

^7^Rwanda Environment Management Authority, Kigali, Rwanda

^8^Department of Mathematics, Faculty of Natural Sciences, Imperial College London, London, UK

^9^Department of Epidemiology and Biostatistics, School of Public Health, Imperial College London, London, UK

^10^Regional Institute for Population Studies, University of Ghana, Accra, Ghana

^11^Abdul Latif Jameel Institute for Disease and Emergency Analytics, Imperial College London, London, UK

^12^MRC Centre for Environment and Health, School of Public Health, Imperial College London, London, UK

^13^Imperial Global Ghana Hub, Accra, Ghana

* Corresponding Author

**Overview**

Kigali monitoring campaign was launched in November 2022 and followed a measurement protocol implemented in Accra, Ghana. During the campaign, we installed 10 ‘fixed’ (yearlong) sites across the city to monitor seasonal variations of noise levels. In addition, we installed a network of 120 ‘rotating’ (weeklong) sites throughout the city, including in all three administrative districts: Gasabo. Kicukiro and Nyarugenge.

**
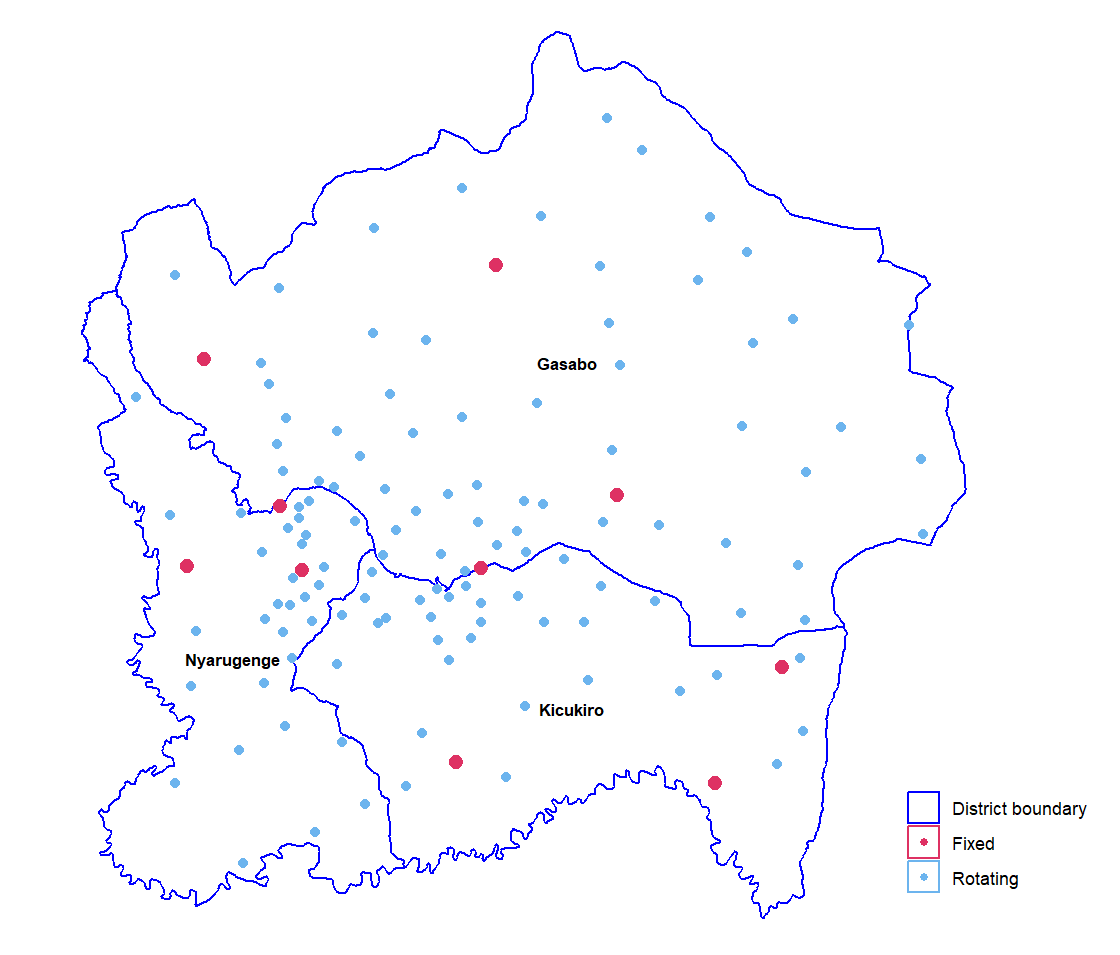
**

**Figure S1: Monitoring sites across the three administrative districts of Kigali city: Gasabo, Kicukiro and Nyarugenge.**

**Descriptive analysis of noise levels for fixed sites**

During the monitoring campaign, we installed 10 fixed (yearlong) sites to monitor seasonal and long-term variations of urban noise pollution. As shown in Table S1, AIMS and NBG sites recorded the highest noise levels across all noise metrics (LAeq_1hr_, LAeq_24hr_, L_day_, and L_night_). The highest median daily (LAeq_24hr_) levels 69.7 (69.1, 70.3) dBA observed at AIMS sites whereas lowest levels at JL and CMU. The highest levels at AIMS and NBG sites are due to traffic as the sites were close to the road and car station whose operate 24/7 hours. The IRday and IRnight were very low at AIMS and very high at MSK, NDB, and GHGM sites.

**Table S1:** Noise metrics and events for 10 fixed (yearlong) sites. Descriptive statistics of noise levels are expressed as medians and interquartile ranges (IQR).

| **Site ID and Noise Metrics/events** | **Minute-by-minute samples** | **LAeq_1hr_ (dBA)** | **LAeq_24hr_ (dBA)** | **L_day_ (dBA)** | **L_night_  (dBA)** | **IR_day_ (%)** | **IR_night_ (%)** |
| --- | --- | --- | --- | --- | --- | --- | --- |
| JL | 568745 | 41.1 (37.1, 44.6) | 45.0 (42.0, 49.6) | 46.5 (43.4, 51.1) | 37.2 (35.8, 39.1) | 56.8 (42.7, 72.8) | 45.8 (36.7, 55.3) |
| CMU | 509104 | 44.8 (43.0, 48.6) | 50.2 (47.3, 54.8) | 51.6 (48.4, 56.4) | 43.5 (42.7, 44.7) | 67.1 (51.4, 80.1) | 24.8 (19.2, 35.2) |
| RSH | 529002 | 48.4 (43.5, 52.0) | 51.4 (49.3, 54.2) | 52.7 (50.6, 55.4) | 45.3 (43.5, 47.2) | 59.7 (52.4, 67.7) | 67.6 (57.3, 74.6) |
| MSK | 566547 | 50.9 (47.5, 54.8) | 53.6 (52.0, 56.2) | 55.0 (53.3, 57.4) | 47.8 (46.7, 49.9) | 67.5 (46.3, 74.8) | 74.7 (66.2, 81.7) |
| AIMS | 541969 | 69.8 (66.9, 71.0) | 69.7 (69.1, 70.3) | 70.9 (70.2, 71.5) | 66.6 (66.0, 67.3) | 9.54 (7.51, 13.9) | 34.8 (27.2, 38.6) |
| NDB | 480354 | 50.7 (44.2, 53.5) | 53.2 (51.8, 55.8) | 54.8 (53.5, 57.4) | 43.3 (42.3, 45.1) | 60.5 (53.8, 72.3) | 74.3 (64.1, 79.0) |
| NBG | 459254 | 68.4 (60.5, 70.2) | 68.5 (67.6, 69.1) | 70.1 (69.3, 70.7) | 61.3 (60.6, 62.1) | 25.8 (22.1, 31.4) | 51.9 (47.6, 55.8) |
| KMHZ | 488849 | 56.4 (53.4, 58.0) | 57.1 (56.2, 58.3) | 58.1 (57.2, 59.3) | 53.8 (53.0, 54.7) | 31.9 (24.6, 42.3) | 46.6 (41.3, 52.4) |
| KRM | 517817 | 55.2 (49.3, 59.0) | 57.8 (56.6, 59.8) | 59.6 (58.3, 61.7) | 48.9 (47.7, 50.1) | 45.3 (38.1, 54.9) | 54.6 (49.6, 60.3) |
| GHGM | 493313 | 63.6 (56.8, 67.5) | 66.1 (63.6, 68.1) | 67.9 (65.2, 69.9) | 56.4 (55.5, 57.5) | 31.0 (20.5, 41.9) | 50.1 (45.4, 54.1) |

*LAeq_1hr_*: A-weighted equivalent continuous 1-h noise level;

*LAeq_24hr_*: A-weighted equivalent continuous 24-h noise level;

*L_day_* & *L_night_*: A-weighted equivalent continuous noise level in the day and night-time;

*IR_day_* & *IR_night_*: Day- and night-time intermittency ratio.**\**

**Temporal variation of noise levels**

The figure below present hourly variation of noise levels across the 10 fixed/yearlong sites. Timeseries shows that AIMS and NBG sites had elevated levels. AIMS is next to a major road with constant traffic, while NBG is beside Nyabugogo bus station, the busiest national bus station in Rwanda, with heavy traffic and loudspeaker use.

**
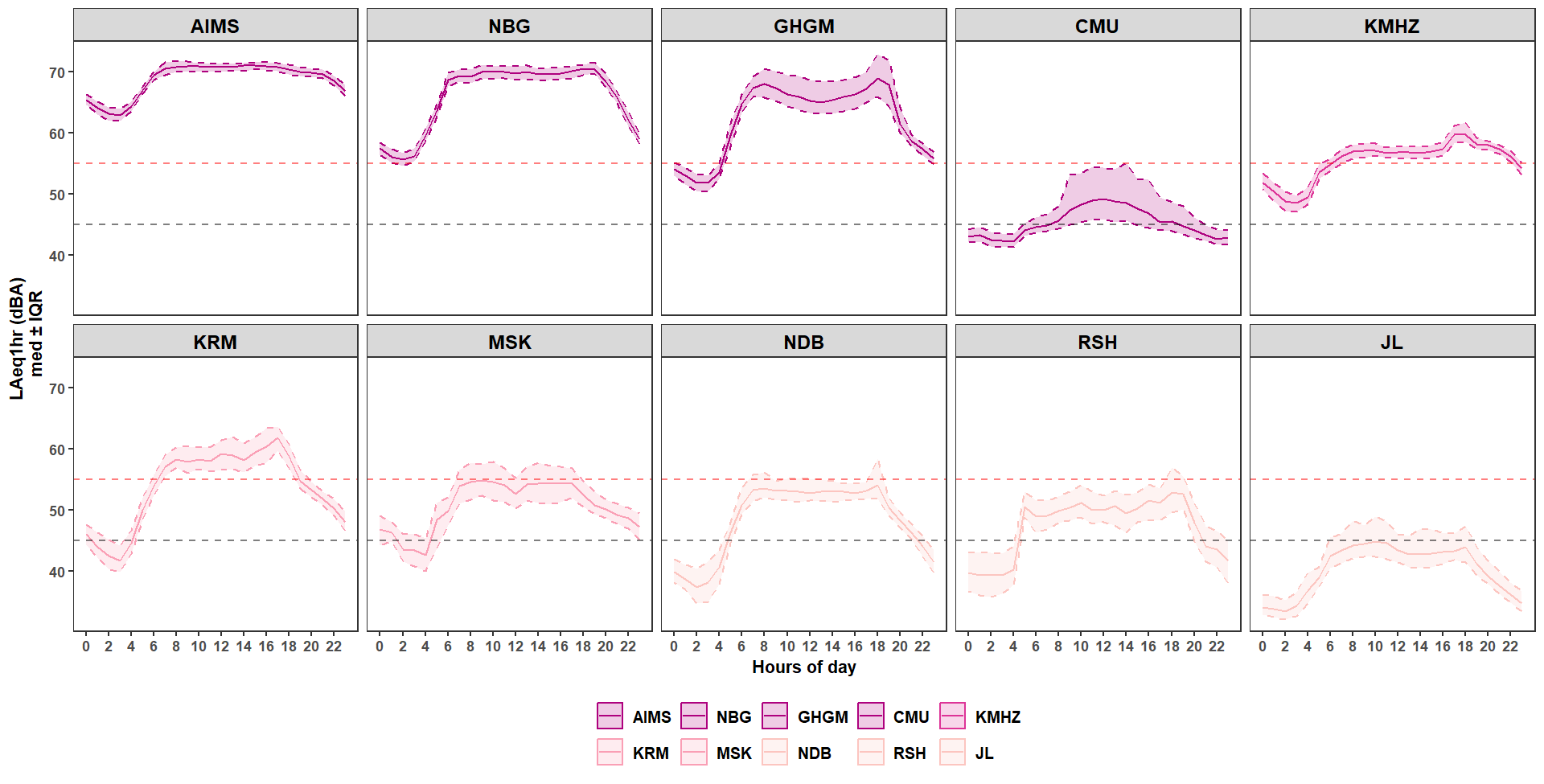
**

**Figure S2:** Hourly noise levels (LAeq_1hr_) across 10 fixed (yearlong) sites. The horizontal dashed red and black lines are the national noise limits for residential areas: 55 dBA for day-time and 45 dBA at night-time.

Like Figure 6 (in main document), fixed sites showed little difference between weekdays and weekend. Higher daytime levels are due to urban activities such as traffic, commercial operations and social activity, while nighttime levels were consistently lower as a results of little influence of human activities.

**
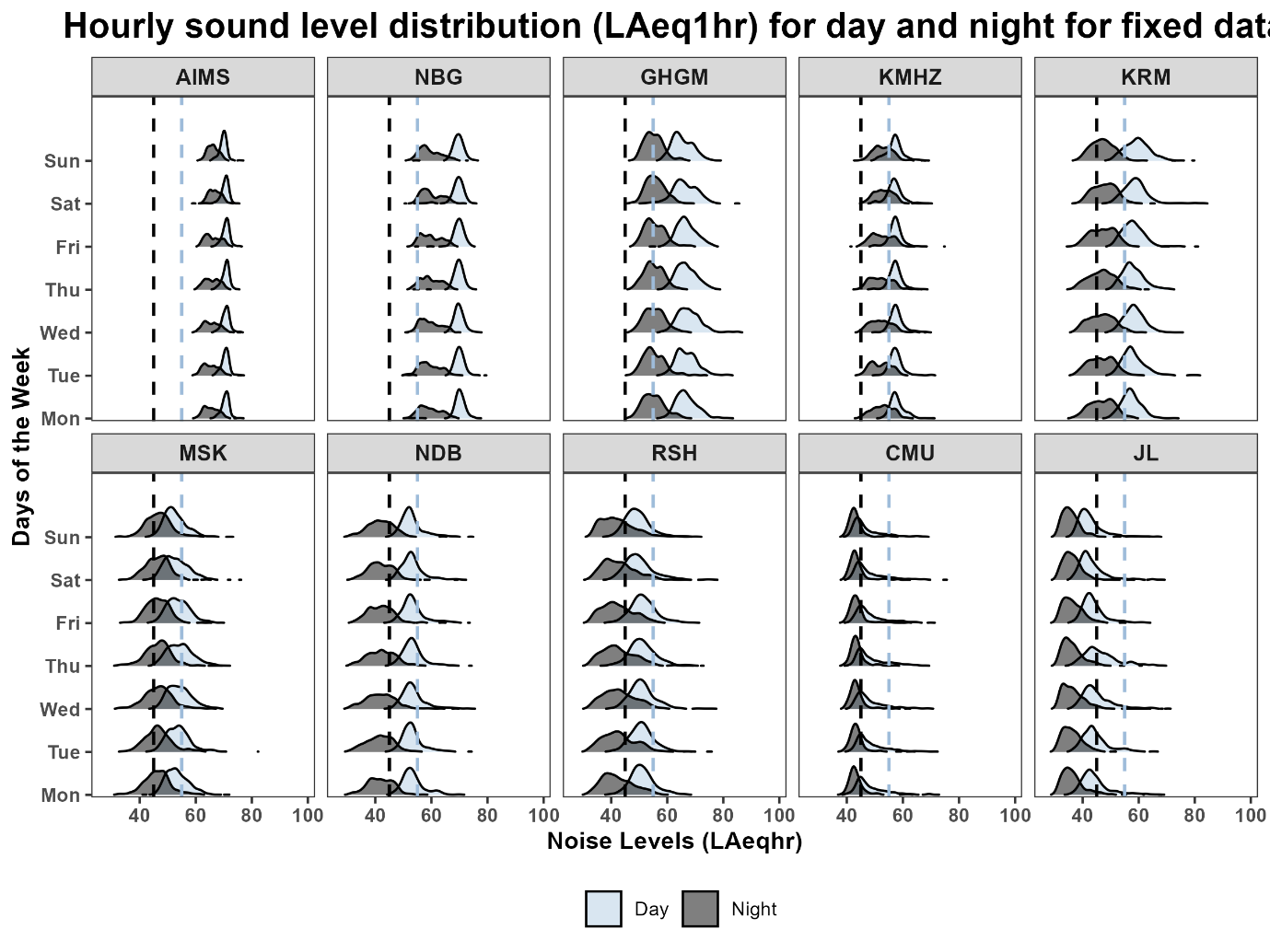
**

**Figure S3**: Distribution of day and night-time hourly noise levels **(**LAeq_1hr_**)** across 10 fixed sites on days of the week. Daytime is defined as 06:00-20:59 and nighttime as 21:00-05:59 according to Rwanda Standard. The yellow and green dashed lines represent nighttime (45 dBA) and daytime (55 dBA) national permissible limit for residential areas, respectively.

**Distribution of Noise Levels (**LAeq_1hr_**)**

The following Figure S4 and S5 show the distribution of noise levels for each land use category and at which time period levels are much produced. The CDF plots show that traffic-dominated sites such as AIMS and NBG consistently experience the highest levels during morning (06:00–09:59) and evening (18:00–20:59) peak hours. BG sites like JL exhibits lower levels.

**
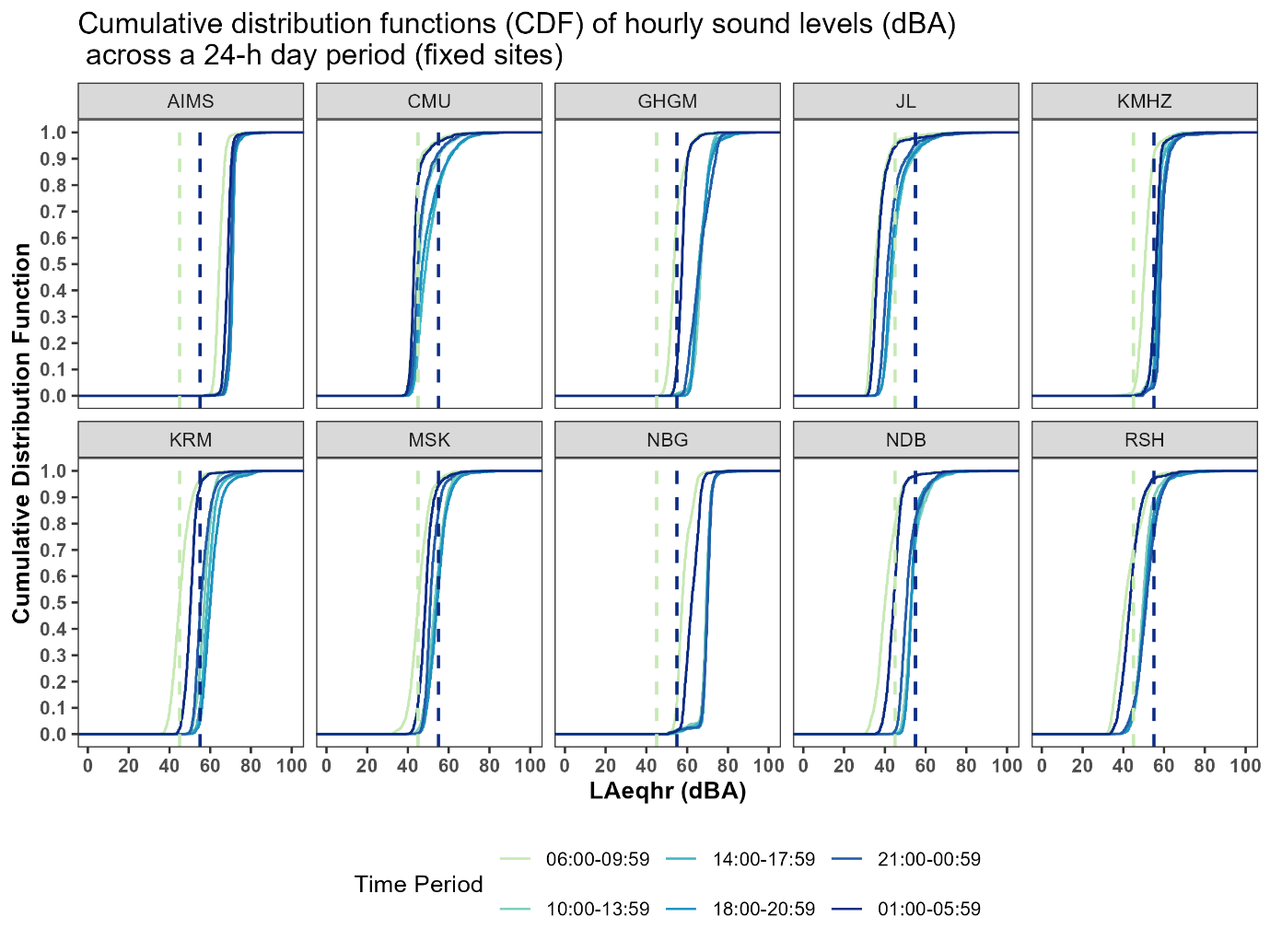
**

**Figure S4: The cumulative distribution functions (CDFs) of hourly noise levels across a 24-hour period for fixed measurement sites.** The dashed lines represent nighttime (45 dBA) and daytime (55 dBA) national permissible limit for residential areas, respectively.

**
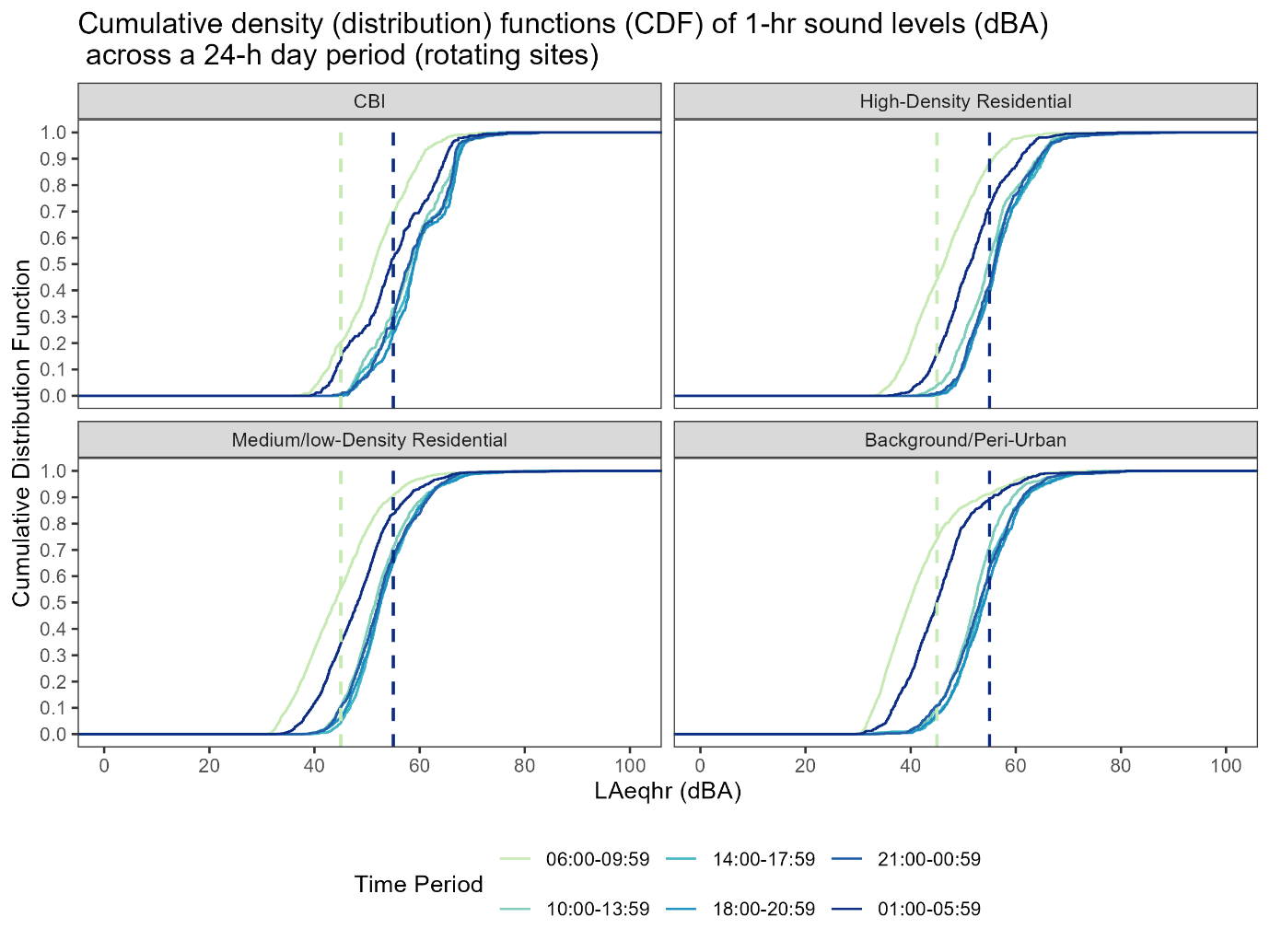
**

**Figure S5: The cumulative distribution functions (CDFs) of 1-hour noise levels (LAeq1hr) across different land use types and time periods for rotating sites**. The dashed lines represent nighttime (45 dBA) and daytime (55 dBA) national permissible limit for residential areas, respectively.

**Analysis of duplicates data**

The median and mean difference between duplicate measurements (Laeq_1min_) are -0.021 dBA and 0.034 dBA whereas the median and mean absolute differences between duplicate measurements were 0.89 dBA and 1.30 dBA respectively. Compared to the noise measurements taken in Accra, Ghana, the median and mean absolute differences for the measurements in Kigali are approximately twice as large as those in Ghana. The median and mean difference for Ghana is 0.74 dBA, and 1.27 dBA respectively ^28^. Figure S6 shows correlation between duplicate measurements against main sites summarized to daily (LAeq_24hr_).

Table S2: Median and Mean differences between 1-min duplicate and main site measurements.

| Laeqmin difference between duplicate measurements (n=332507, minute-by-minute samples) | |
| --- | --- |
| Median | -0.02090576 |
| Mean | 0.03373576 |
| Absolute difference between and main duplicate measurement (n=332507, minute-by-minute samples) | |
| Median | 0.8955383 |
| Mean | 1.296712 |

**
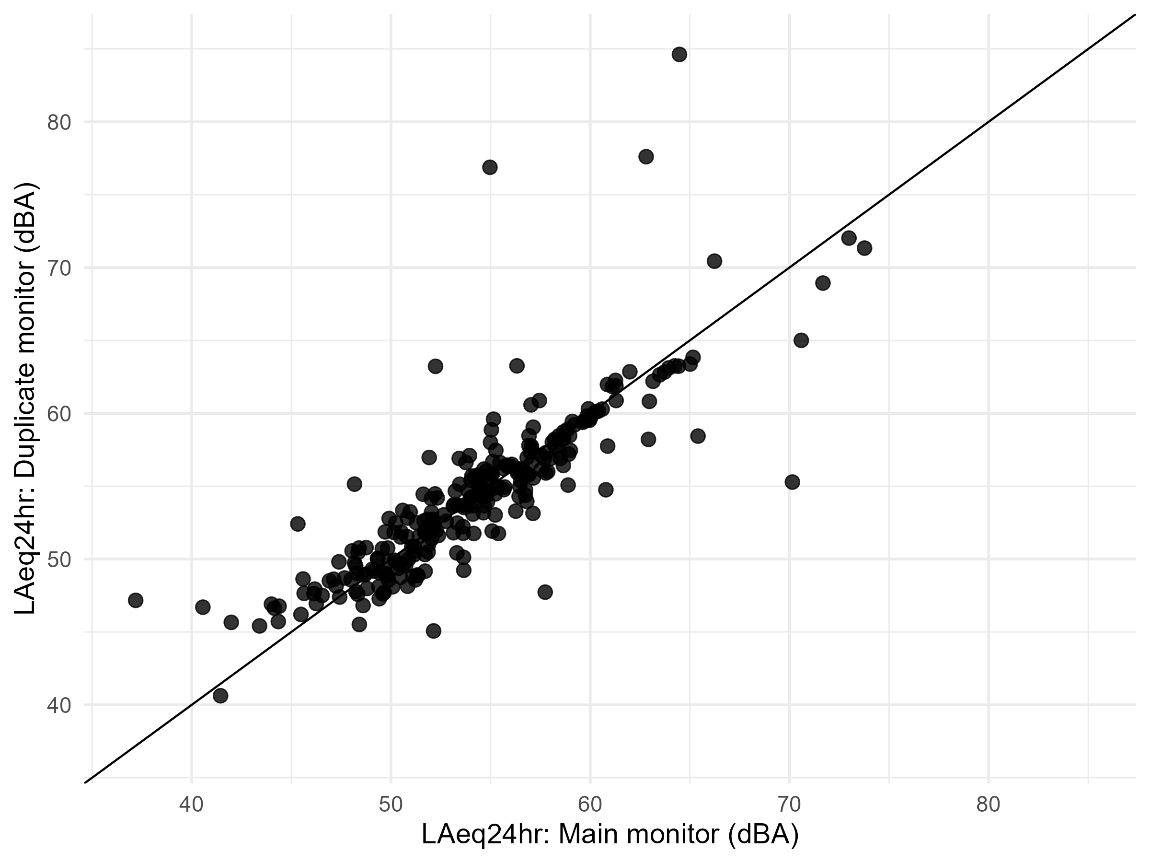
**

**Figure S6: Scatterplot between the median daily (LAeq_24hr) noise levels for main and duplicate monitors conducted at 33.59% of rotating sites.** ID: measurement site names.

Figure S7 exhibits the distribution of day and night-time hourly noise levels (LAeq1hr) on 10 fixed (yearlong) sites across the months of the year. Throughout the months of the years, the majority of fixed sites had relatively higher day-time noise levels due to urban activities, particularly at traffic and commercial sites. By considering the seasonal variation, little variations were noted, with traffic-heavy sites showing fluctuations, and despite lower nighttime noise, urban areas still experience significant residual noise.


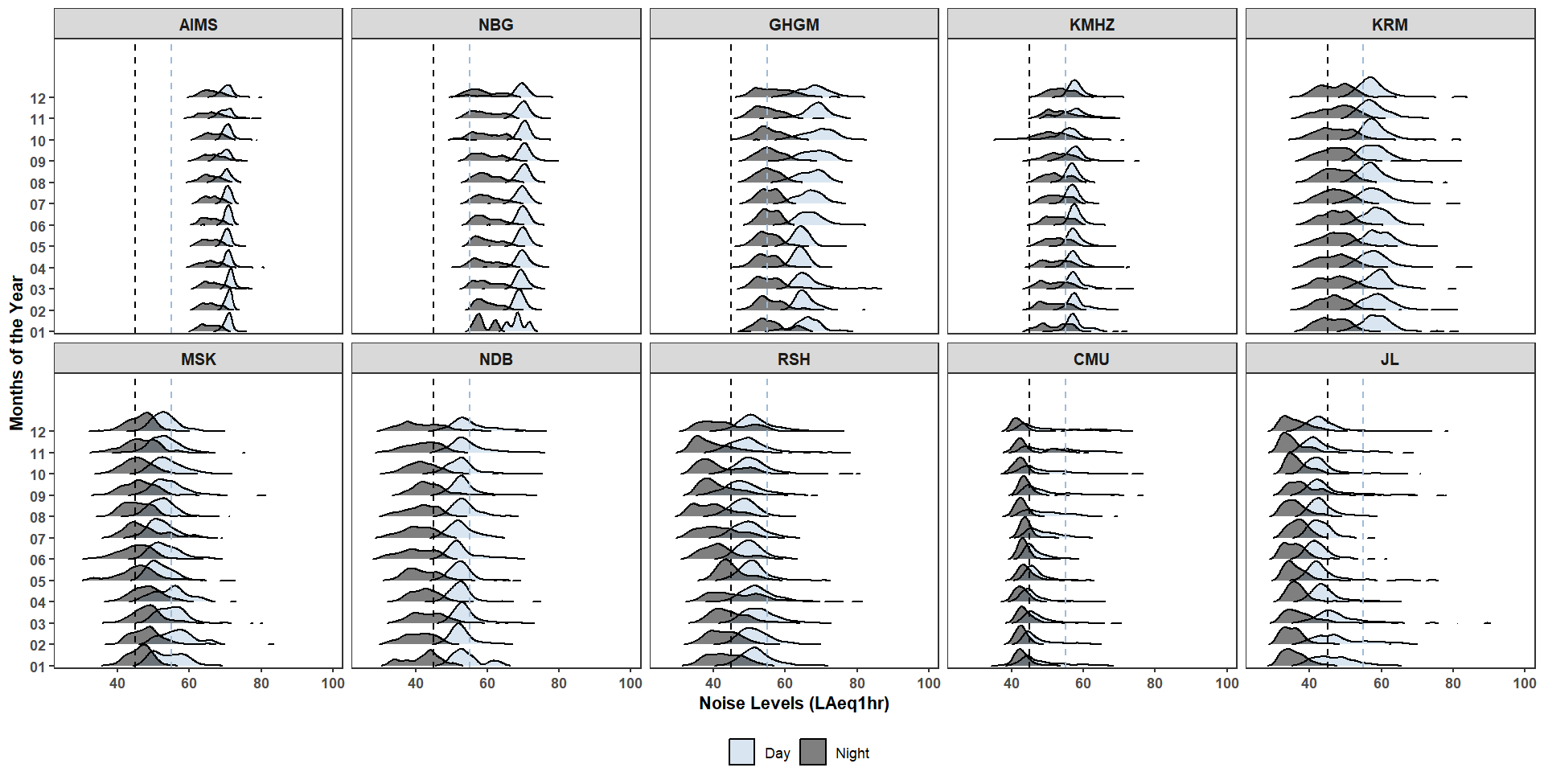


**Figure S7: Distribution of day and night-time hourly noise levels (LAeq_1hr_) across 10 fixed sites throughout the year**. Daytime is defined as 06:00-20:59 and nighttime as 21:00-05:59 according to Rwanda Standard. The purple and light blue dashed lines represent nighttime (45 dBA) and daytime (55 dBA) national permissible limit for residential areas, respectively.

Table S3: The site-specific noise data and corresponding sample sizes (N) used for statistical summaries.

|  | **LAeq_1hr_** | **LAeq_24hr_** |
| --- | --- | --- |
| All rotating sites (120) | | |
| *Background/Peri-Urban (BG): (31)* | 5113 | 245 |
| *Medium/low-density residential (LD): (47)* | 7590 | 367 |
| *High-density residential (HD): (27)* | 4451 | 216 |
| *Commercial/Business/Industrial (CBI): (15)* | 2660 | 127 |
| All fixed sites (10) | | |
| *Background/Peri-Urban (BG): (3)* | 26326 | 1108 |
| *Medium/low-density residential (LD): (2)* | 18100 | 765 |
| *High-density residential (HD): (1)* | 8157 | 344 |
| *Commercial/Business/Industrial (CBI): (4)* | 33431 | 1409 |
